# Do Children (and Adults) Benefit From a Prediction Error Boost in One-Shot Word Learning?

**DOI:** 10.5334/joc.342

**Published:** 2024-01-12

**Authors:** Chiara Gambi, Jaroslaw R. Lelonkiewicz, Davide Crepaldi

**Affiliations:** 1 Cardiff University, UK; 2 University of Warwick, UK; 3 Scuola Internazionale Superiore di Studi Avanzati (SISSA), Italy

**Keywords:** prediction error, mutual exclusivity, disconfirmed predictions, memory retention, word learning, language acquisition

## Abstract

Influential theories and computational models suggest error-based learning plays an important role in language acquisition: Children learn new words by generating predictions about upcoming utterances and revising those predictions when they are erroneous. Critically, revising stronger (rather than weaker) predictions should further enhance learning. Although previously demonstrated in adults, such prediction error boost has not been conclusively shown in children. To close this gap, we tested 107 participants between the ages of 5 and 10. We found little evidence that word learning in this age group benefits from a prediction error boost. Moreover, we also failed to replicate previous evidence for such an effect in adults. Based on a detailed task analysis, we suggest the variation in adult findings may be partly explained by differences in encoding strategies and that, relatedly, the protracted development of the episodic memory system might explain why children do not experience robust benefits from having stronger (rather than weaker) predictions disconfirmed.

The speed with which children add words to their vocabulary depends not only on their environment (e.g., Romeo et al., 2018), but also on their language processing skills – that is, how efficiently they process language in real time (Fernald & Marchman, 2012; Gambi, Jindal, et al., 2021). Computational and experimental studies (Chang et al., 2006; Havron et al., 2019; Ramscar, Dye, & McCauley, 2013; Ramscar et al., 2010; Reuter et al., 2019) suggest that one particular skill – predict-and-revise – may be key to early vocabulary acquisition. Children may learn by generating predictions about upcoming utterances and by revising such predictions when they are disconfirmed by the language they actually hear. New words are of course particularly likely to disconfirm predictions, and so it seems plausible that predict-and-revise could play a role in lexical acquisition. However, there is little evidence that this mechanism contributes to word learning during early childhood (up to age 5). In this paper, we present new developmental data showing it is unlikely predict-and-revise contributes to word learning even in middle childhood (age 5 to 10). To foreshadow our conclusion, we argue that existing evidence – and the new data reported here – together call into question the idea that a predict-and-revise mechanism might explain rapid word learning over the preschool and primary school years.

Predict-and-revise refers to the ability to anticipate upcoming input and rapidly update one’s expectations when they do not match the observed input (Reuter et al., 2019). According to error-based accounts of language acquisition, children keep track of associations between cues (e.g., the presence of a particular object) and outcomes (e.g., the occurrence of a particular linguistic form) to learn about various aspects of the language system they are exposed to (Ramscar, 2021), including word-object associations (Ramscar, Dye, & Klein, 2013; Ramscar et al., 2010), morphology (Ramscar, Dye, & McCauley, 2013), and syntax (Fazekas et al., 2020; Peter et al., 2015). When they encounter cues associated with a particular outcome, children predict the occurrence of that outcome. But crucially, when the predicted outcome does not occur, an error signal is produced, causing the associative weights between cues and outcomes to be adjusted – it is this adjustment that underlies children’s learning.

One hypothesis that stems from these accounts is that a larger error signal should be accompanied by stronger learning, because a greater mismatch between predicted and observed input should cause a larger adjustment to associative weights. In other words, larger prediction errors should have larger effects on learning. While not all theoretical accounts of error-based learning formulate this hypothesis explicitly (for a recent discussion, see Babineau et al., 2022), surprisal effects in structural priming studies are usually interpreted as supporting it. Structural priming refers to the tendency to re-use a sentence structure one has just heard (Messenger et al., 2022). Crucially, this tendency is amplified for structures that are unexpected. For example, children experience stronger structural priming following sentences such as *The zookeeper brings the giraffe some food* as compared to *The zookeeper gives the giraffe some food*. This is because in English child-directed speech this sentence structure (direct object or DO) is much less likely to occur with the verb *bring* than *give* (Peter et al., 2015). Stronger priming after a *bring* sentence than a *give* sentence is explained as a consequence of the fact that children were more surprised by the occurrence of the DO with *bring* (larger prediction error) than by the occurrence of the DO with *give* (smaller or no prediction error). Greater structural priming – i.e., an increased tendency to re-use the DO structure, following a *bring*-DO prime than a *give*-DO prime, is thus interpreted as evidence for the effect of prediction error on learning (Fazekas et al., 2020; Peter et al., 2015).

Specifically, surprisal effects in structural priming show that the magnitude of prediction error - and its effect on learning - depend on the strength of preceding expectations (for a theoretical justification outside of language see Henson & Gagnepain, 2010; Quent et al., 2021; Rescorla & Wagner, 1972). The reason a DO is more surprising following *bring* than *give* is that – though exposure to language input – English-learning children have developed a (stronger) expectation that *bring* will be used in a prepositional object (PO) structure (e.g., *The zookeeper brings some food to the giraffe*). When they encounter *bring* used in a DO structure during the experiment, the larger prediction error causes a larger increase in the level of activation of the DO structure and in the weight of the connection between the verb *bring* and the DO structure (see Chang et al., 2006 for a formalization of this idea in a computational model) – this change in activation/weights represents learning of structural knowledge (for a child encountering the DO with *bring* for the first time) or fine-tuning of existing knowledge to the local input statistics (i.e., “DO with *bring* is more common than I previously thought”). It is also the mechanism underlying priming and explains why participants become more likely to reuse the DO structure (both with *bring*, and with other verbs) immediately after a *bring* prime than a *give* prime.

Here we ask whether the hypothesis that stronger predictions – when disconfirmed - lead to greater learning also applies to the acquisition of novel words. New words, by virtue of being unfamiliar, mismatch our expectations and generate an error signal that should trigger a long-lasting revision to linguistic knowledge. Crucially, this signal should be larger (and the learning stronger) when the new word occurs after a different (familiar) word was strongly predicted, compared to when no word was particularly expected. We refer to this advantage in word learning as the *prediction error boost* (see Greve et al., 2017 for an empirical investigation in adults outside the domain of language learning).

Two previous studies manipulated the strength of linguistic predictions to test whether young children indeed benefit from a prediction error boost when acquiring new vocabulary (Gambi, Pickering, et al., 2021; Reuter et al., 2019), but the evidence has been inconclusive. Reuter et al. (2019) showed that 3-to-5 year olds with more advanced predict-and-revise skills are better at word learning: Children who heard a novel word following a sentence context that was highly predictive of a different, familiar word, showed better learning of the association between the novel word and an unfamiliar object to the extent that they (first) strongly predicted the familiar word and (then) rapidly revised that expectation (as revealed by eye-tracking). However, this study also showed that – overall – children’s memory for novel word-object associations was *worse* for words that disconfirmed a stronger compared to a weaker linguistic expectation – the opposite of what should happen according to error-based learning accounts. Note that both highly predictive and non-predictive trials induced some prediction error, as they all ended in a novel word which, by definition, children could not have predicted, but the magnitude of this prediction error was hypothesed to be larger following the highly predictive trials.

Thus, Reuter et al.’s findings are not entirely consistent with the hypothesis that larger prediction errors lead to better word learning. One possibility is that children sometimes fail to revise their expectations: Children who generate particularly strong predictions may not experience any prediction error, and instead associate the novel word to a familiar object (effectively, treating it as a synonym for the expected familiar word; Babineau et al., 2022).

A recent study by Gambi, Pickering, et al. (2021) explicitly controlled for this possibility. In this study, two large samples (both ~80) of 2-to-4 year olds were also exposed to sentences that were either highly predictive or non-predictive of a familiar word, but ended with a novel word. This study collected data on children’s explicit referent choices (though it did not collect eye-tracking data) so it was possible to identify trials on which children associated the novel word to the familiar, rather than the novel, object. Crucially, even after removing such trials - on which participants had failed to revise their predictions - there was still no evidence for a prediction error boost in children; in contrast, adults – who were also tested using the same materials - showed this effect precisely as hypothesised by error-based learning accounts. Note that this difference between children and adults could not be explained by the fact that the task was too difficult for children. In fact, as long as children selected the novel object as referent for the novel word during the learning phase of the study, they showed above chance performance during test. Finally, there was no indication that children predicted less strongly than adults in the first place, as both children and adults showed different choice behaviour in the learning phase depending on whether the sentence was highly predictive of the familiar object or non-predictive.

In sum, when taken together, Reuter et al. (2019) and Gambi, Pickering, et al. (2021) do not provide unambiguous evidence that larger prediction errors lead to better word learning in children, while Gambi, Pickering, et al. (2021) shows that adults do. Why is this the case? We suggest that one important factor that has so far been overlooked is the protracted development of the episodic memory system. Learning new words ultimately requires storing conceptual and phonological representations in long-term memory, but the initial stages of word learning rely heavily on episodic encoding via the hippocampus (Davis & Gaskell, 2009). The hippocampus is responsible for encoding and retrieving detailed memories about specific encounters with particular items (including the context in which those items were encountered; e.g., Rugg et al., 2012), and is sensitive to novelty (Duszkiewicz et al., 2019; Shohamy & Adcock, 2010) and unexpectedness (Gruber et al., 2018). Hence, hippocampal involvement is particularly important in one-shot learning tasks – such as the ones used by Reuter et al. (2019) and Gambi, Pickering, et al. (2021) – where participants encounter novel words just once in contexts that make their occurrence more vs. less expected (as we describe in more detail below, *The current study)*.

Importantly, hippocampal development is protracted (Gómez & Edgin, 2016), and episodic memory matures well into middle childhood (i.e., between 6 and 11 years of age; Ghetti & Bunge, 2012; Lee et al., 2014; Newcombe, 2015). Episodic memory for items in context – that is, the ability to remember that a specific item was encountered in a particular context – is especially slow to develop (Lee et al., 2016), as is the ability to use semantic memory (memory about facts and concepts, generalised over multiple events) in episodic memory tasks (Ghetti & Bunge, 2012). Both of these memory skills may be pre-requisites for the emergence of a prediction error boost in word learning. Semantic memory (e.g., knowledge about the semantic restrictions imposed by some verbs on their arguments) allows the generation of predictions based on familiar aspects of the context a novel word appears in, while the ability to associate a word to its context allows the memory representation for a new word to be strengthened when it disconfirms a contextual prediction.

Thus, an immature episodic memory system could explain why previous work has failed to find clear evidence for the prediction error boost in early childhood. In support of this, children younger than 5 do not benefit from incorrect guessing in errorful generation tasks, in contrast to older children (Carneiro et al., 2018; cf. Faran et al., 2017). Furthermore, while children above the age of 8 show the so-called hypercorrection effect – i.e., the tendency to better remember the correct answer to questions that were initially answered incorrectly with greater rather than lower confidence (Metcalfe & Finn, 2012) – other work shows that disconfirmed predictions only boost learning in those 9–12 year olds with more advanced executive function skills (Brod et al., 2019), possibly because strong inhibitory control is required to suppress an incorrect guess.

In sum, the memory literature suggests that children may only begin to show a prediction error boost in word learning during middle childhood. In this study, we tested children between the ages of 5 and 10. If error-based learning accounts of word acquisition are correct, we would expect even the youngest children in this age range to show a prediction error boost. In contrast, a later developmental onset for this effect would call into question the idea that error-based learning is a fundamental mechanism in early word acquisition.

## The current study

We adapted the one-shot word learning task of Gambi, Pickering, et al. (2021; Experiment 4). In this task, participants encountered novel words (e.g., *cheem*), each presented only once, and learnt to associate them to unfamiliar objects without explicit instruction. On each trial, participants were shown two candidate referents for a novel word: One unfamiliar, nameless object and one familiar, easy-to-name object. Both adults and children typically infer that the novel word must refer to the nameless object (Halberda, 2003). Crucially, prediction strength was manipulated by presenting novel words at the end of sentences which, given the candidate pictures on the screen, either did or did not encourage a strong expectation for a particular word. For example, participants heard *Now, Peppa will eat the…* either while looking at pictures of an apple and an unfamiliar, but seemingly edible object or while looking at pictures of a car and that same unfamiliar object. Apple is a likely candidate object for the verb *eat*, while car is an unlikely object for this verb. Therefore, participants had a much stronger expectation that the familiar object would be mentioned when this object was an apple compared to when it was a car. Following this learning phase, participants’ memory for the association between each novel word and the corresponding unfamiliar object was tested (see *Methods*).

In this task, participants generate predictions based on their knowledge of verb-event structure (e.g., knowledge of the kind of entities that can serve as the object of the verb *eat*), which is part of semantic memory (McRae et al., 1997), but they also need to associate the novel word to the novel object. Given they only encounter each word-object pair once, they must rely on episodic memory (Cooper et al., 2019; Remon et al., 2020). Importantly, it is likely that participants’ relatively good performance (~70% to 80% for adults learning 8 words; Gambi, Pickering, et al., 2021; Experiments 1–4) is supported by binding the novel word to the familiar context (i.e., the sentence and the familiar object). Indeed, we know that adults deploy existing semantic knowledge when learning words during incidental reading (Mak et al., 2021), and that constraining sentence contexts enable fast learning of the meaning of novel words (Borovsky et al., 2010).

In our task, when participants’ memory is tested after learning, hearing a novel word likely triggers retrieval of the sentence and familiar object that were presented with it during learning, and this in turn could mediate retrieval of the correct unfamiliar object. In this way, the familiar aspects of the context – whose representations in semantic memory are much stronger – could facilitate learning of the association between a novel word and a novel object. Crucially, when the combined linguistic and visual context generates a stronger prediction for a particular word form, which is then disconfirmed by the occurrence of the novel word, the binding between the familiar context and the novel word form will be stronger (Quent et al., 2021), explaining the prediction error boost effect found in adults.

The critical novelty of the current study is that it involved children between the ages of 5 and 10, allowing us to investigate the developmental trajectory of prediction error in word acquisition and its links to memory. Our sample consisted of Italian primary school children. Because of this, we had to translate and adapt the English materials used by Gambi, Pickering, et al. (2021). In addition, we tested 2 samples of Italian-speaking adults (one in the lab, one online) to check if Gambi, Pickering, et al.’s (2021) findings for English adults would replicate with a new set of materials.

## Open Science Statement

This study was not pre-registered. All materials, code, data and analyses for this project can be found at: https://osf.io/yct9p/.

## Methods

### Participants

We tested 107 children between the ages of 5 and 10. Participants were recruited through primary schools in and around Trieste, Italy, and were tested at Scuola Internazionale Superiore di Studi Avanzati (SISSA) during an educational event (Brains at Work 2018/2019). Since children attended the event in their school grades, we set our recruitment targets by grade. In the Italian system, primary school spans five grades, with most children starting between the ages of 5 and 6 and leaving between the ages of 10 and 11.

Initially, we aimed to recruit 20 children per grade (grades 1-4 as these took part in the educational event) to reach an overall sample size of 80, as in Gambi, Pickering, et al. (2021). Power calculations indicated that a sample of 80 participants would achieve 83% power. These calculations were based on the adult data from Gambi, Pickering, et al. (2021), and so did not account for the possibility that effect sizes may be smaller and variability higher in child samples (we return to this potential limitation in the *Results* section).

Due to recruitment constraints, we oversampled from higher grades and undersampled from the first grade (grade 1: 10 children, grade 2: 26, grade 3: 30, and grade 4: 40; for one child grade information was missing). In the Italian system each grade spans adjacent ages, so we decided to split children into three age groups that were somewhat more balanced in terms of sample size. The final sample comprised of 29 children who were 5-to-6 year old (Mage = 75.5 months, range [62–83], 13 females), 30 7-year-olds (Mage = 89.4 months, range [84 – 95], 15 females), and 48 8-to-10-year-olds (Mage = 103.7 months, range [96–121], 26 females). Note that our analyses tested for the prediction error boost across the entire sample, as well as investigating differences between age groups.

We had information about the child’s spoken languages for 86 children: All were native speakers of Italian, and 21 spoke at least one other language. Caregivers reported that three children (one 6-year-old and two 7-year-olds) had been diagnosed with a developmental disorder (language delay, ADHD, articulation disorder). No children were excluded from the analyses reported below.

Two samples of Italian-speaking adults also completed the study. The first group (N = 68, Mage = 24.4 years, range [19, 34], 48 females) did so in the lab, while the second (N = 58; Mage = 23.5 years, range [17^1^ 34], 29 females; one participant did not provide age information) was tested online (see Gambi, Pickering, et al., 2021 for details of the power analysis that suggested a sample size of at least 58 participants). Participants in the lab study were recruited through a database available at SISSA and paid €10/hour. Participants in the online study were recruited through Prolific Academic and paid £6.96/hour. All lab-based participants and all but one online participant reported to be native speakers of Italian (one participant was a native speaker of Punjabi with Italian as an additional language). Seventy-three participants reported English as their only additional language, one reported Ukrainian as their only additional language, while a further 28 participants had two or more additional languages. Language profiles are reported for completeness, but since Gambi, Pickering, et al. (2021) found no indication that bilingual/multilingual speakers perform differently from monolinguals in one-shot word learning, we disregard this factor in the analyses below.

The study received ethical approval from SISSA (child and lab-based adult study) and the University of Cardiff (online adult study). Informed consent was collected prior to the study. For child participants, consent forms were sent to the families who expressed interest in the educational event several days in advance. Parental/legal guardians were advised to seek consent from children prior to signing on their behalf. In addition, the experimenters monitored children for consent throughout the testing session. For adult participants, consent was collected at the beginning of the testing session via a paper (laboratory group) or digital (on-line group) form.

### Materials and Procedure

**Figure 1:**
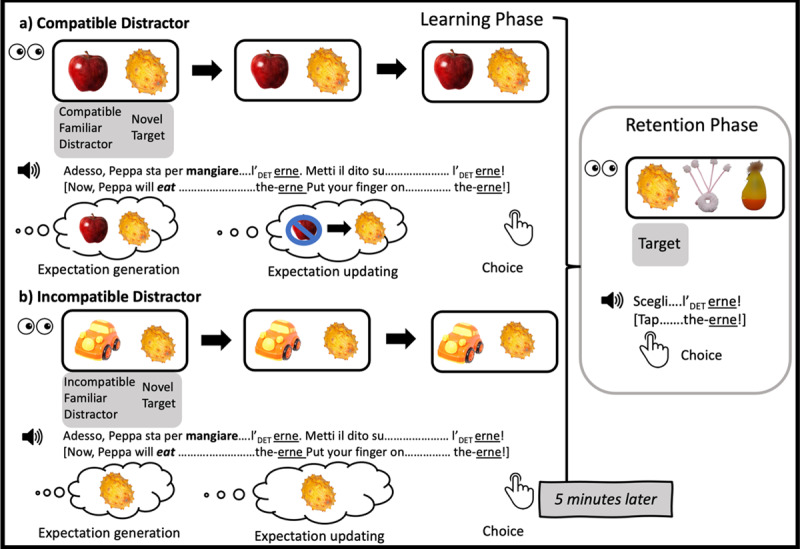
Schematic depiction of design, procedure and participant mental operations on different types of learning trials used in this study. Panel **(a)** illustrates Compatible Distractor trials, panel **(b)** Incompatible Distractor trials. In this depiction, we assume the participant chose the novel object as the referent of the novel word during the learning phase. (This figure was adapted with permission from Gambi, Pickering, et al., 2021.)

Materials and procedure were matched as closely as possible to Gambi, Pickering, et al. (2021, Experiment 4 and 7). We used the same experimental design (see Figure 1), stimulus lists and counterbalancing. The spoken sentences were translated from English into Italian and re-recorded by a female native speaker of Italian using child-directed prosody; we also created a new set of novel words, following Italian phonotactic constraints.

Participants were exposed to novel words embedded in sentences that always contained a semantically constraining verb, such as *mangiare/eat* in *Adesso, Peppa sta per mangiare…/Now, Peppa will eat…*[NOVEL WORD]. Unfamiliar target objects were always compatible with the semantic restrictions of this verb (e.g., the unfamiliar object paired with the sentence in the example above was an exotic fruit). Listeners’ expectations were manipulated by varying the identity of the familiar distractor object. Familiar objects either fitted the semantic constraint of the verb (e.g., an edible object, such as an apple, for the sentence above) or did not (e.g., a non-edible object, such as a car, for the same sentence). Verb-compatible familiar objects are plausible candidates for prediction, while verb-incompatible familiar objects are not. Thus, listeners should be more likely to generate (incorrect) expectations in the compatible familiar object than the incompatible familiar object condition.

The experiment consisted of two phases: A learning phase and a retention phase, separated by a 5–10 minute break. During the learning phase, participants encountered 8 novel pseudowords and 8 novel (unfamiliar) objects (one per trial). In addition, they completed 2 practice trials at the start, and 4 filler trials which were randomly interspersed with experimental trials. All learning trials started with participants clicking or tapping on a picture of the cartoon character Peppa Pig, displayed at the top of the screen. This triggered a pre-recorded sentence. Participants could listen to the recording as many times as they wished, but rarely did so more than once. They then heard a pre-recorded instruction to select one of the photographs on the bottom half of the screen (*Scegli/Choose* [NOVEL WORD]), which depicted one familiar and one unfamiliar object. The trial ended once the participants chose one of the two objects. On half the experimental trials the familiar object was compatible with the meaning of the verb, while on the other half it was not (see Figure 1); compatible and incompatible distractor trials were randomly intermixed with each other and with filler trials (cf. Reuter et al., 2019 where the manipulation was blocked). Distractor compatibility was manipulated within participants and items, counterbalanced across two lists. Filler sentences were always predictive of (and ended with) the name of the familiar object (*e.g., Questa volta, Peppa sta per cullare… il bimbo/In this one, Peppa will rock… the baby*) to encourage participants to predict familiar words.

We used 8 pseudowords: *erne, intre, angre, utte, uepe, obe, umbe, alse*. Like their English equivalents in Gambi, Pickering, et al. (2021), they were 2-to-4 phonemes long. Italian and English pseudowords did not differ significantly in neighbourhood density based on OLD20 (i.e., the average number of edits necessary to turn one word into another calculated for the 20 closest orthographic neighbors in the relevant – Italian or English – lexicon; Yarkoni et al., 2008). We used SUBTLEX-UK (Van Heuven et al., 2014) for the English lexicon and SUBTLEX-IT (Crepaldi et al., 2015) for the Italian lexicon. English pseudowords were all monosyllabic, whereas Italian pseudowords were disyllabic (reflecting the fact that Italian words tend to be longer). Italian pseudowords began with a vowel (which meant they could be preceded by the gender-ambiguous determiner /l/, grapheme *l’*) and ended in /e/, which is compatible with both masculine and feminine gender. Thus, it was not possible to use gender-marking on the article to constrain prediction of the upcoming noun (Ito et al., 2020). A full list of Italian items and their English translations can be found on the OSF repository for this project (*experimental_materials* folder, file name: Italian_and_English_stimuli_upload.xlsx).

Target words were recorded separately together with the preceding determiner and combined with the spoken sentence contexts online, so that we could fully randomize object-word pairings for each participant. Trial order was randomized separately for each participant and phase of the experiment (learning and retention). The learning phase was completed first for all items, so that learning and retention trials were fully blocked, with no interleaving.

Following completion of the learning phase, children completed a series of tapping games involving familiar cartoon characters; adults watched a short video from an Italian episode of Peppa Pig and answered four comprehension questions (to ensure they were paying attention). Finally, participants completed 8 retention trials (see Figure 1). They tapped/clicked on Peppa Pig (top of the screen), which triggered a pre-recorded instruction (e.g., *Scegli/Tap* [NOVEL WORD]). The bottom half of the screen displayed three photographs in random order: The target object (i.e., the unfamiliar object that had appeared on the learning trial the novel word was used on) and two distractor objects. One distractor was a target object from a different experimental trial, while the other had been used on a filler trial (i.e., it had not been named and was therefore never a target). Across trials, each unfamiliar target object appeared twice (once as target, once as distractor) and each unfamiliar filler object also appeared twice (always as a distractor, but paired with two different words). Participants did not receive feedback about the accuracy of their choices.

Children and lab-based adult participants completed the task on a desktop PC while wearing headphones. Adults were tested individually and completed this task after a statistical learning task that lasted around 30 minutes (Lelonkiewicz et al., 2023). Children were tested individually, accompanied by an experimenter. Online adult participants had to pass a stringent test to ensure they were wearing headphones and were completing the experiment in a quiet environment (adapted from: https://github.com/ChaitLabUCL/HeadphoneCheck_Test; Milne et al., 2021).

The task was custom-coded in HTML and Javascript. Please email the corresponding author for an OSF link to the code: Some of the visual stimuli we used are protected by copyright, so we are unfortunately unable to provide the link here, but we welcome requests to share with individual researchers.

### Data analysis

Since our dependent variables are choice data, we used generalised linear mixed-effects models (function *glmer* from the *lme4* package, version 1.1–23) with a logistic link function (Bates et al., 2015) in R (R Development Core Team, Version 3.5.1). Random effect structure was maximal, except when correlations between random effects or higher-order random slopes had to be dropped to aid convergence. Fixed effects were contrast-coded and centered. Estimated random factors are included in model output tables. As well as *p* values, we report 95% confidence intervals for model estimates from the *confint* function (method = Wald). These tables were generated using the *tab_model* function from the *sjPlot* package (sjPlot, Version 2.6.3). Full model specifications are available at the OSF link (*analyses* folder, file name: *Italian_pred_summary.Rmd*).

Participants’ choices on learning trials (i.e., choosing the novel vs. familiar object) and their accuracy on retention trials (i.e., choosing the target vs. one of the two distractor objects) were analysed as a function of Distractor compatibility (compatible vs. incompatible distractor, with the latter being the reference level). For retention trials, accuracy was coded in terms of whether participants were able to retain the pairing of the novel label with the novel object, regardless of whether they had chosen the novel object or the familiar distractor during the learning phase. However, the analyses of participants’ choices on retention trials controlled for this by including Choice at learning in the model structure (i.e., choosing the novel object vs. familiar distractor on the corresponding learning trial).

We expected to replicate the findings of Gambi, Pickering, et al. (2021) and show that children are more likely to correctly recall the association between a novel object and a novel word if they had explicitly selected the novel object as the referent for the novel word during the learning phase (as opposed to selecting the familiar object). Since Choice at learning never interacted with Distractor compatibility in any of the models, we did not conduct separate analyses looking only at trials for which the novel referent had been chosen during learning. We were primarily interested in testing the effect of distractor compatibility on retention accuracy – i.e., whether there is a prediction error boost effect, and the extent to which this effect changes with the age of the participant.

We first analysed data from all age groups together (omnibus analysis). Age was included as an additional categorical predictor in these analyses, with 4 levels: 5–6 year olds, 7 year olds, 8–10 year olds, and adults (adult data from the lab-based and online studies were combined into a single adult age group category). The Age categorical predictor was coded as 3 backward difference contrasts. The first contrast compared children aged 7 to children aged 5-6; the second contrast compared children aged 8–10 to children aged 7, and the third and final contrast compared adults to 8–10 year olds. The models also included interactions between the three age contrasts and the other predictors (and their interactions).

When this omnibus analysis revealed some significant interactions with Age, we followed it up with separate analyses for each age group. Because we had 4 different age groups, the significance threshold for these follow-up analyses was conservatively set to .05/4 = .0125. Finally, we also conducted a follow-up analysis with age (in months) as a continuous (centred) variable (on child data only).

## Results

### Learning phase

We first analyse participants’ choices during the learning phase to check whether our manipulation of distractor compatibility was successful. Distractor compatibility influenced children’s and adults’ choices alike: When the familiar distractor object was compatible with the semantic restrictions of the verb, participants of all ages were more likely to map the novel word to an object for which they already knew a name, presumably because they interpreted the novel label as a subordinate-level name (e.g., a type of apple) or a synonym of the familiar word (see Table 1). Accordingly, the main effect of Distractor compatibility was significant (*p* <. 001; see Table S1 in supplementary materials) and there were no differences between age groups either in the overall likelihood of selecting the unfamiliar object (across conditions) or in the size of the Distractor compatibility effect (all *p*’s >= .076; see Table S1).

**Table 1:** Learning phase: Unfamiliar object choice (%) in the learning phase by Distractor compatibility condition in each age group.


AGE GROUP	N	DISTRACTOR COMPATIBILITY	UNFAMILIAR OBJECT CHOICE (%)

5–6 year olds	29	incompatible	95

		compatible	75

7 year olds	30	incompatible	95

		compatible	78

8–10 year olds	48	incompatible	95

		compatible	76

Adults	126	incompatible	95

		compatible	85


### Retention phase

**Figure 2:**
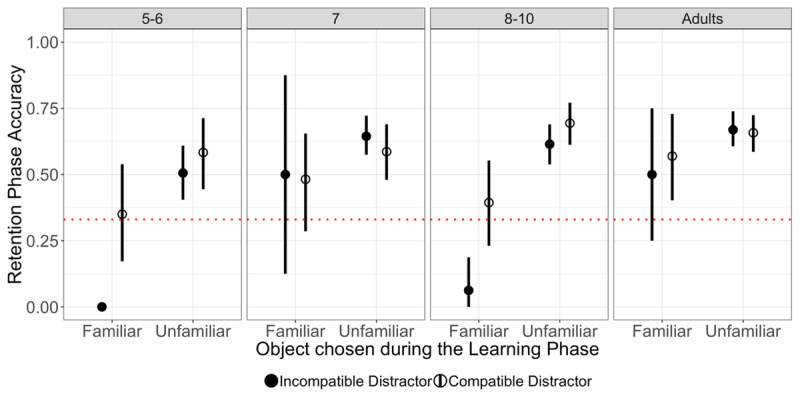
Retention accuracy (%) as a function of Distractor compatibility and of the referent chosen during learning (Familiar vs. Unfamiliar). Prediction error was smaller in the incompatible distractor (filled circle) than in the compatible distractor (empty circle) conditions. The error bars represent 95% bootstrap CI’s (1000 samples) over subjects. When 5-to-6 year olds chose the familiar object (i.e., the incompatible distractor) on incompatible distractor trials during learning, they never selected the correct target at retention, hence no CI is displayed for this condition with mean accuracy = 0. The error bars are generally larger for familiar object choices because these choices were much rarer and therefore these conditions are represented by fewer data points (see Table 1). The dashed horizontal lines represent chance performance (33% given on each retention trial participants chose from three objects).

Unsurprisingly, memory for the novel word-object associations was better (regardless of condition) in adults than 8–10 year olds (*p* = .015); there were no significant differences across younger and older children (both *p*’s >= .682; see Table 2 and Table S2 in Supplementary Materials). As can be seen in Table 2 and Figure 2, participants in all age groups were well above chance (33%) in the retention phase when they had selected the unfamiliar object during the learning phase. Note that our models cannot test for the comparison to chance directly, and this conclusion is based on visual inspection of the figure and confidence intervals. We cannot draw firm conclusions about overall performance when participants had selected the familiar object during the learning phase as the number of trials in these conditions was much smaller (as indicated by the much wider confidence intervals in Figure 2).

**Table 2:** Retention phase: Target (unfamiliar) object choice at retention (accuracy) (%) by Distractor compatibility and by Choice at learning (i.e., the choice of object during the learning phase), separately for each age group.


AGE GROUP	N	DISTRACTOR COMPATIBILITY	CHOICE AT LEARNING	TARGET OBJECT CHOICE AT RETENTION (ACCURACY) (%)

5–6 year olds	29	incompatible	Familiar	0

			Unfamiliar	49

		compatible	Familiar	45

			Unfamiliar	59

7 year olds	30	incompatible	Familiar	50

			Unfamiliar	65

		compatible	Familiar	52

			Unfamiliar	57

8–10 year olds	48	incompatible	Familiar	10

			Unfamiliar	60

		compatible	Familiar	36

			Unfamiliar	70

Adults	126	incompatible	Familiar	48

			Unfamiliar	70

		compatible	Familiar	63

			Unfamiliar	70


Most importantly, Distractor compatibility did not affect the likelihood of choosing the correct target for a novel word (i.e., no evidence for prediction error boost across ages; *p* = .669), nor did the choice of image made during the learning phase (*p* = .654). There was also no interaction between Distractor compatibility and Choice at learning (*p* = .728), which may have indicated that the prediction error boost effect was present only when participants selected the unfamiliar object as the novel word referent during the learning phase (as observed in Experiments 1–3 of Gambi, Pickering, et al., 2021). However, since Age interacted with Distractor compatibility and with Choice at learning, we followed up the omnibus analysis with separate analysis by age group (significant interactions are highlighted in bold in Table S2). Specifically, the interactions suggested that the prediction error boost effect was greater in 8–10 year olds than in 7 year olds (*p* = .045) and that the memory advantage for words that had been explicitly mapped onto the unfamiliar object during the learning phase was greater in 8–10 year olds than in 7 year olds (*p* = .026), and smaller in adults than in 8–10 year olds (*p* = .018). We found no differences between 5–6 year olds and 7 year olds (see Table S2). Finally, there were no three-way interactions between Distractor compatibility, Choice at learning, and Age (see Table S2).

Follow-up analyses by age group (see Table S3 in Supplementary Materials), however, did not provide strong evidence for a prediction error boost in any age group, not even adults (all *p*’s >= .034; to account for multiple comparisons, we used a Bonferroni-adjusted alpha threshold of .0125). The choice made during learning had an effect in 8–10 year olds (*p* < .001) and adults (*p* = .008), but not in younger children (all *p*’s > .027). The model for 5–6 year olds did not include an interaction between Distractor compatibility and the choice made during learning due to convergence issues; for all other age groups, the models included this interaction but it was not significant (all *p*’s >= .141).

In sum, we found no evidence for a prediction error boost in children aged 5 to 10. Additional analyses of the child data including the child’s age (in months) as a (centred) continuous predictor were consistent with these conclusions (see Supplementary Materials, Section 4).

### Pooled analyses of Italian participants (this study) and English participants (Gambi, Pickering, et al., 2021)

While the weak evidence reported above suggests that the prediction error boost effect is unlikely to play a key role in early word learning, it is possible that our analyses for children were underpowered, because of large between-participant variability and/or due to the limited number of items (Westfall et al., 2014). Therefore, we conducted some additional analyses pooling together data from this study and from the English study of Gambi, Pickering, et al. (2021). Aside from the language difference (see details of the adaptation to Italian in the Materials and Procedure section above) and the fact that Italian children were older (see Participants), note that all Italian participants were tested on the same version of the study that was used in Experiment 4 of Gambi, Pickering, et al. (2021) with adults and in Experiment 7 of Gambi, Pickering, et al. (2021) with 2-to-4 year olds. In addition, Gambi, Pickering, et al. (2021) also tested some English adults on an identical design but using different stimuli lists (Experiment 5) and both English adults (Experiment 1–3) and English 2-to-4 year olds (Experiment 6) on a different design that varied the content of the sentence (as in Reuter et al., 2019) rather than the identity of the familiar distractor object in order to manipulate prediction error (see Gambi, Pickering, et al., 2021 for full details).

First we pooled data from all children in both studies (N = 166 in Gambi, Pickering, et al., 2021 and N = 107 in the current study), regardless of which experiment version they completed. In this pooled analysis, the effect of prediction error on word learning emerged as small at best (Log odds = 0.33, corresponding to an odds ratio of 1.39, *p* = .024; see Supplemental Materials, Section 9). Moreover, our pooled model (see Supplemental Materials, Section 9) shows by-participant variability in the prediction error boost effect was much larger (.24) compared to by-item variability (estimated at 0 and thus removed from the model), which indicates the low number of items is unlikely to have greatly impacted power. Finally, we also computed the Bayes Factor for the prediction error boost (see Supplemental Materials, Section 10) and found only anecdotal change in evidence (Jeffreys, 1939; as cited in Schad et al., 2022) for an effect of prediction error on retention accuracy in children.

As for adults, we pooled together data from those experiments that used a comparable manipulation of prediction error (Experiments 4 and 5 in Gambi, Pickering, et al., 2021 and the current study). These analyses confirmed that the magnitude of the prediction error boost was larger in the English than the Italian samples (*p* = .010; see Supplementary Materials, Section 5), suggesting a reliable difference between studies (though the Bayes Factor for this interaction indicated only anecdotal evidence in the favour of a difference; see Supplementary Materials, Section 10). Importantly, Distractor compatibility affected adult choices during *learning* to a similar extent across studies (see Supplemental Materials, Section 6), showing it was not the case Italian sentences simply failed to bias adults’ expectations. Finally, performance on attention checkers and overall retention accuracy were also comparable across English and Italian samples, making it unlikely that Italian participants were simply less attentive during the task (see Supplemental Materials, Section 7).

## Discussion

While error-based learning has been proposed as a fundamental mechanism in language acquisition, previous empirical work with children up to the age of 5 was inconclusive regarding its role in novel word learning (Gambi, Pickering, et al., 2021; Reuter et al., 2019). In this study, we tested older children on a paradigm closely modelled on previous work, and found very little evidence that *even in middle childhood* children are more likely to remember the association between a novel word and a novel object when they have encountered them under conditions that generated a larger prediction error signal.

We acknowledge that current data are still insufficient to precisely pinpoint the developmental onset of the effect, but clearly they do not provide strong support for theories that propose a fundamental role for prediction error in *early* word learning (Chang et al., 2006; Ramscar, 2021). By the age of 5, not only is word learning well underway, but children have also developed very sophisticated linguistic prediction and revision skills (Gambi, Jindal, et al., 2021; Havron et al., 2019; Babineau et al., 2022). Thus, they should clearly benefit from the prediction error boost if indeed the process of word learning is driven by prediction error.

In contrast, the effect of prediction error on word learning appears small and variable. We suggest this is because it fundamentally relies on a mature episodic memory system which is not in place until later in childhood (Ghetti & Bunge, 2012). Specifically, we suggest that one-shot word learning is dependent on binding a novel word-object pair to familiar aspects of the linguistic and visual context: For example, remembering the association between the word *cheem* in the familiar sentence frame *Now, Peppa will eat the cheem* and an unfamiliar exotic fruit benefits from the association to the familiar verb *eat* in the sentence and from the presence of a familiar edible object (e.g., an apple) in the visual context. Indeed, there is evidence that providing semantic information during word learning helps memory in 5-to-9 year olds (Henderson et al., 2013), and that adults deploy existing semantic knowledge when learning novel words (Borovsky et al., 2010; Mak et al., 2021; Lelonkiewicz et al., 2023).

However, binding novel information to context and using long-term semantic knowledge to scaffold episodic memory are both relatively advanced skills (Ghetti & Bunge, 2012; Lee et al., 2016). The late development of these skills may explain why only the older children in our sample showed emerging evidence of a prediction error boost in word learning. Interestingly, overall memory performance improved only minimally between the ages of 5 and 10; this suggests that, while a fully-developed episodic memory system may be necessary to benefit from predictive contextual representations, other, earlier-developing memory structures are sufficient to perform well above chance (33%) in one-shot word learning (Picard et al., 2012).

One important limitation to our findings is the failure to replicate the prediction error boost effect in Italian adults, which may suggest some issue with our adaptation of the original English materials (note that Gambi, Pickering, et al., 2021 replicated the effect in adults across 5 experiments). That said, our Italian materials were equally successful as the English materials in biasing participants’ expectations. Therefore, it is more likely that the difference in findings is due to differences in how Italian-speaking and English-speaking adults approached the task.

One possibility is that Italian adults distributed their attention *differently* during learning trials. While Italian adults’ memory was sensitive to the choice of referent made during the learning phase, English adults were equally good at retention regardless of the choice they had made during learning. Specifically, when Italian adults chose the familiar object as the referent of the novel word during learning, they were then less likely to choose the correct unfamiliar target object during the retention phase. This suggests they did not retain information about potential alternative referents for the novel word once they made the decision to associate it to one of the referents on the screen (see Trueswell et al., 2013).

In other words, Italian adults likely adopted a narrow focus of attention during learning. This might explain why they were also less likely to benefit from the prediction error boost, as this is dependent upon binding the novel word and unfamiliar object to familiar aspects of the linguistic and visual context (with the binding being reinforced when a stronger expectation is disconfirmed). While this explanation remains highly speculative, in exploratory analyses we found some preliminary evidence that the magnitude of the prediction error boost was greater for those Italian adults who were affected *less* by the choice they made during the learning phase (*r_s_* = –.215, *p* = .021; see Supplemental materials, Section 8).

Is it possible that children - who showed no evidence of a prediction error boost effect – also adopted a narrow focus of attention? If so, we would expect young children to also show a strong effect of learning choice on retention accuracy, which they did in the English data of Gambi, Pickering, et al. (2021). However, Italian 7-year-olds showed neither a prediction error boost effect nor an effect of learning choice on retention accuracy, while 8-to-10 year olds showed both. Thus it is unclear at the moment whether children cannot effectively bind the novel word to the context, while (at least some) adults do.

To conclude, our findings do not support the idea that a predict-and-revise learning mechanism plays a significant role in the early acquisition of the lexicon. They also highlight potentially significant variability in how both children and adults approach word learning tasks. Future work should ask when in development children become capable of generating detailed enough expectations at the relevant linguistic level, and of revising those expectations when the input indicates they are incorrect, and, critically, which memory system(s) allow for efficient error-based learning.

## Data Accessibility Statements

Author Note: All materials, code, data and analyses for this project can be found at: https://osf.io/yct9p/.

## Additional File

The additional file for this article can be found as follows:

10.5334/joc.342.s1 Supplementary Materials. Full model outputs, detailed descriptions and results for additional analyses, including using age as a continuous predictor, comparing Italian and Englidh samples, and Bayesian analyses.

## Footnotes

One participant was below the age of 18 when tested, but we were unaware of this and the study was advertised as requiring a minimum age of 18.

## References

[1] Babineau, M., Havron, N., Dautriche, I., de Carvalho, A., & Christophe, A. (2022). Learning to predict and predicting to learn: Before and beyond the syntactic bootstrapper. Language acquisition, 1–24. DOI: 10.1080/10489223.2022.207821135281590

[2] Bates, D., Maechler, M., Bolker, B., & Walker, S. (2015). Fitting linear mixed-effects models using lme4. Journal of Statistical Software, 67(1), 1–48. DOI: 10.18637/jss.v067.i01

[3] Borovsky, A., Kutas, M., & Elman, J. (2010). Learning to use words: Event-related potentials index single-shot contextual word learning. Cognition, 116(2), 289–296. DOI: 10.1016/j.cognition.2010.05.004 20621846 PMC2904319

[4] Brod, G., Breitwieser, J., Hasselhorn, M., & Bunge, S. A. (2019). Being proven wrong elicits learning in children–but only in those with higher executive function skills. Developmental science, e12916. DOI: 10.1111/desc.12916 31626721

[5] Carneiro, P., Lapa, A., & Finn, B. (2018). The effect of unsuccessful retrieval on children’s subsequent learning. Journal of Experimental Child Psychology, 166, 400–420. DOI: 10.1016/j.jecp.2017.09.010 29032207

[6] Chang, F., Dell, G. S., & Bock, K. (2006). Becoming syntactic. Psychological Review, 113(2), 234–272. DOI: 10.1037/0033-295X.113.2.234 16637761

[7] Cooper, E., Greve, A., & Henson, R. N. (2019). Little evidence for Fast Mapping (FM) in adults: A review and discussion. Cognitive neuroscience, 10(4), 196–209. DOI: 10.1080/17588928.2018.1542376 30451079 PMC6711760

[8] Crepaldi, D., Amenta, S., Mandera, P., Keuleers, E., & Brysbaert, M. (2015). SUBTLEX-IT. Subtitle-based word frequency estimates for Italian Annual Meeting of the Italian Association for Experimental Psychology, Rovereto. https://lrlac.sissa.it/publications/subtlex-it-subtitle-based-word-frequency-estimates-italian

[9] Davis, M. H., & Gaskell, M. G. (2009). A complementary systems account of word learning: neural and behavioural evidence. Philosophical Transactions of the Royal Society B: Biological Sciences, 364(1536), 3773–3800. DOI: 10.1098/rstb.2009.0111 PMC2846311 19933145

[10] Duszkiewicz, A. J., McNamara, C. G., Takeuchi, T., & Genzel, L. (2019). Novelty and dopaminergic modulation of memory persistence: a tale of two systems. Trends in Neurosciences, 42(2), 102–114. DOI: 10.1016/j.tins.2018.10.002 30455050 PMC6352318

[11] Faran, Y., Osher, Y., Sofen, Y., & Shalom, D. B. (2017). Errorful and errorless learning in preschoolers: at what age does the errorful advantage appear? Cognitive development, 44, 150–156. DOI: 10.1016/j.cogdev.2017.10.002

[12] Fazekas, J., Jessop, A., Pine, J., & Rowland, C. (2020). Do children learn from their prediction mistakes? A registered report evaluating error-based theories of language acquisition. Royal Society open science, 7(11), 180877. DOI: 10.1098/rsos.180877 33391776 PMC7735343

[13] Fernald, A., & Marchman, V. A. (2012). Individual differences in lexical processing at 18 months predict vocabulary growth in typically developing and late-talking toddlers. Child Development, 83(1), 203–222. DOI: 10.1111/j.1467-8624.2011.01692.x 22172209 PMC3266972

[14] Gambi, C., Jindal, P., Sharpe, S., Pickering, M. J., & Rabagliati, H. (2021). The relation between preschoolers’ vocabulary development and their ability to predict and recognize words. Child Development, 92(3), 1048–1066. DOI: 10.1111/cdev.13465 32865231

[15] Gambi, C., Pickering, M.J., & Rabagliati, H. (2021). Prediction error boosts retention of novel words in adults but not in children. Cognition, 211, 104650. DOI: 10.1016/j.cognition.2021.104650 33721717

[16] Ghetti, S., & Bunge, S. A. (2012). Neural changes underlying the development of episodic memory during middle childhood. Developmental cognitive neuroscience, 2(4), 381–395. DOI: 10.1016/j.dcn.2012.05.002 22770728 PMC3545705

[17] Gómez, R. L., & Edgin, J. O. (2016). The extended trajectory of hippocampal development: Implications for early memory development and disorder. Developmental cognitive neuroscience, 18, 57–69. DOI: 10.1016/j.dcn.2015.08.009 26437910 PMC4808499

[18] Greve, A., Cooper, E., Kaula, A., Anderson, M. C., & Henson, R. (2017). Does prediction error drive one-shot declarative learning? Journal of Memory and Language, 94, 149–165. DOI: 10.1016/j.jml.2016.11.001 28579691 PMC5381756

[19] Gruber, M. J., Hsieh, L.-T., Staresina, B. P., Elger, C. E., Fell, J., Axmacher, N., & Ranganath, C. (2018). Theta phase synchronization between the human hippocampus and prefrontal cortex increases during encoding of unexpected information: a case study. Journal of Cognitive Neuroscience, 30(11), 1646–1656. DOI: 10.1162/jocn_a_01302 29952700

[20] Halberda, J. (2003). The development of a word-learning strategy. Cognition, 87(1), B23–B34. DOI: 10.1016/S0010-0277(02)00186-5 12499109

[21] Havron, N., de Carvalho, A., Fiévet, A. C., & Christophe, A. (2019). Three-to four-year-old children rapidly adapt their predictions and use them to learn novel word meanings. Child Development, 90(1), 82–90. DOI: 10.1111/cdev.13113 30004578

[22] Henderson, L., Weighall, A., & Gaskell, G. (2013). Learning new vocabulary during childhood: Effects of semantic training on lexical consolidation and integration. Journal of Experimental Child Psychology, 116(3), 572–592. DOI: 10.1016/j.jecp.2013.07.004 23981272

[23] Henson, R. N., & Gagnepain, P. (2010). Predictive, interactive multiple memory systems. Hippocampus, 20(11), 1315–1326. DOI: 10.1002/hipo.20857 20928831

[24] Ito, A., Gambi, C., Pickering, M. J., Fuellenbach, K., & Husband, E. M. (2020). Prediction of phonological and gender information: An event-related potential study in Italian. Neuropsychologia, 136, 107291. DOI: 10.1016/j.neuropsychologia.2019.107291 31805283

[25] Jeffreys, H. (1939). Theory of probability. Oxford: Clarendon Press.

[26] Lee, J. K., Ekstrom, A. D., & Ghetti, S. (2014). Volume of hippocampal subfields and episodic memory in childhood and adolescence. NeuroImage, 94, 162–171. DOI: 10.1016/j.neuroimage.2014.03.019 24642282

[27] Lee, J. K., Wendelken, C., Bunge, S. A., & Ghetti, S. (2016). A time and place for everything: Developmental differences in the building blocks of episodic memory. Child Development, 87(1), 194–210. DOI: 10.1111/cdev.12447 26493950 PMC4733390

[28] Lelonkiewicz, J. R., Ktori, M., & Crepaldi, D. (2023). Morphemes as letter chunks: Linguistic information enhances the learning of visual regularities. Journal of Memory and Language, 130, 104411. DOI: 10.1016/j.jml.2023.104411

[29] Mak, M. H., Hsiao, Y., & Nation, K. (2021). Anchoring and contextual variation in the early stages of incidental word learning during reading. Journal of Memory and Language, 118, 104203. DOI: 10.1016/j.jml.2020.104203

[30] McRae, K., Ferretti, Todd R., & Liane Amyote, T. R. (1997). Thematic roles as verb-specific concepts. Language and Cognitive Processes, 12(2–3), 137–176. DOI: 10.1080/016909697386835

[31] Messenger, K., Branigan, H., Buckle, L., & Lindsay, L. (2022). How does syntactic priming experience support language development? In K. Messenger (Ed.), Syntactic Priming in Language Acquisition Representations, mechanisms and applications. (pp. 57–82). John Benjamins. DOI: 10.1075/tilar.31.04mes

[32] Metcalfe, J., & Finn, B. (2012). Hypercorrection of high confidence errors in children. Learning and Instruction, 22(4), 253–261. DOI: 10.1016/j.learninstruc.2011.10.004

[33] Milne, A. E., Bianco, R., Poole, K. C., Zhao, S., Oxenham, A. J., Billig, A. J., & Chait, M. (2021). An online headphone screening test based on dichotic pitch. Behavior Research Methods, 53(4), 1551–1562. DOI: 10.3758/s13428-020-01514-0 33300103 PMC7725427

[34] Newcombe, N. S. (2015). Commentary: Memory development: Halfway there? International Journal of Behavioral Development, 39(4), 304–305. DOI: 10.1177/0165025415573647

[35] Peter, M., Chang, F., Pine, J. M., Blything, R., & Rowland, C. F. (2015). When and how do children develop knowledge of verb argument structure? Evidence from verb bias effects in a structural priming task. Journal of Memory and Language, 81, 1–15. DOI: 10.1016/j.jml.2014.12.002

[36] Picard, L., Cousin, S., Guillery-Girard, B., Eustache, F., & Piolino, P. (2012). How do the different components of episodic memory develop? Role of executive functions and short-term feature-binding abilities. Child Development, 83(3), 1037–1050. DOI: 10.1111/j.1467-8624.2012.01736.x 22364311

[37] Quent, J. A., Henson, R. N., & Greve, A. (2021). A predictive account of how novelty influences declarative memory. Neurobiology of Learning and Memory, 179, 107382. DOI: 10.1016/j.nlm.2021.107382 33476747 PMC8024513

[38] R Development Core Team. (Version 3.5.1). [Computer Software]. http://www.R-project.org

[39] Ramscar, M. (2021). A discriminative account of the learning, representation and processing of inflection systems. Language, Cognition and Neuroscience. DOI: 10.1080/23273798.2021.2014062

[40] Ramscar, M., Dye, M., & Klein, J. (2013). Children value informativity over logic in word learning. Psychological Science, 24(6), 1017–1023. DOI: 10.1177/0956797612460691 23610135

[41] Ramscar, M., Dye, M., & McCauley, S. M. (2013). Error and expectation in language learning: The curious absence of mouses in adult speech. Language, 89(4), 760–793. DOI: 10.1353/lan.2013.0068

[42] Ramscar, M., Yarlett, D., Dye, M., Denny, K., & Thorpe, K. (2010). The effects of feature-label-order and their implications for symbolic learning. Cognitive Science, 34(6), 909–957. DOI: 10.1111/j.1551-6709.2009.01092.x 21564239

[43] Remon, D., Loevenbruck, H., Deudon, M., Girardie, O., Bouyer, K., Pascalis, O., & Thorpe, S. (2020). 24-Month-olds and over remember novel object names after a single learning event. Journal of Experimental Child Psychology, 196, 104859. DOI: 10.1016/j.jecp.2020.104859 32408989 PMC7262577

[44] Rescorla, R. A., & Wagner, A. R. (1972). A theory of Pavlovian conditioning: Variations in the effectiveness of reinforcement and nonreinforcement. Classical conditioning II: Current research and theory, 2, 64–99.

[45] Reuter, T., Borovsky, A., & Lew-Wlliams, C. (2019). Predict and redicrect: Prediction errors support children’s word learning. Developmental psychology, 55(8), 1656–1665. DOI: 10.1037/dev0000754 31094555 PMC6876992

[46] Romeo, R. R., Leonard, J. A., Robinson, S. T., West, M. R., Mackey, A. P., Rowe, M. L., & Gabrieli, J. D. (2018). Beyond the 30-Million-Word Gap: Children’s Conversational Exposure Is Associated With Language-Related Brain Function. Psychological Science. DOI: 10.1177/0956797617742725 PMC5945324 29442613

[47] Rugg, M. D., Vilberg, K. L., Mattson, J. T., Sarah, S. Y., Johnson, J. D., & Suzuki, M. (2012). Item memory, context memory and the hippocampus: fMRI evidence. Neuropsychologia, 50(13), 3070–3079. DOI: 10.1016/j.neuropsychologia.2012.06.004 22732490 PMC3472091

[48] Schad, D. J., Nicenboim, B., Bürkner, P. C., Betancourt, M., & Vasishth, S. (2022). Workflow techniques for the robust use of bayes factors. Psychological Methods. Advance online publication. DOI: 10.1037/met0000472 35266787

[49] Shohamy, D., & Adcock, R. A. (2010). Dopamine and adaptive memory. Trends in Cognitive Sciences, 14(10), 464–472. DOI: 10.1016/j.tics.2010.08.002 20829095

[50] SjPlot. (Version 2.6.3). Data Visualization for Statistics in Social Science. In [Computer Software]. Lüdecke, D. https://CRAN.R-project.org/package=sjPlot.

[51] Trueswell, J. C., Medina, T. N., Hafri, A., & Gleitman, L. R. (2013). Propose but verify: Fast mapping meets cross-situational word learning. Cognitive Psychology, 66(1), 126–156. DOI: 10.1016/j.cogpsych.2012.10.001 23142693 PMC3529979

[52] Van Heuven, W. J., Mandera, P., Keuleers, E., & Brysbaert, M. (2014). SUBTLEX-UK: A new and improved word frequency database for British English. Quarterly Journal of Experimental Psychology, 67(6), 1176–1190. DOI: 10.1080/17470218.2013.850521 24417251

[53] Westfall, J., Kenny, D. A., & Judd, C. M. (2014). Statistical power and optimal design in experiments in which samples of participants respond to samples of stimuli. Journal of Experimental Psychology: General, 143(5), 2020–2045. DOI: 10.1037/xge0000014 25111580

[54] Yarkoni, T., Balota, D., & Yap, M. (2008). Moving beyond Coltheart’s N: A new measure of orthographic similarity. Psychonomic Bulletin & Review, 15(5), 971–979. DOI: 10.3758/PBR.15.5.971 18926991

