# Supplementary material for: Do Children (and Adults) Benefit From a Prediction Error Boost in One-Shot Word Learning?
Source: J Cogn. 2024 Jan 12;7(1):13. doi: 10.5334/joc.342 (PMC10785960; doi:10.5334/joc.342)
Supplement: Supplementary Materials. — Full model outputs, detailed descriptions and results for additional analyses, including using age as a continuous predictor, comparing Italian and Englidh samples, and Bayesian analyses. [file joc-7-1-342-s1.pdf]

Supplementary Materials

Section 1. Learning phase – Omnibus analysis – Model summary.

Table S1. Learning phase: Model output for the logistic linear mixed-effects model predicting the likelihood of choosing the unfamiliar object during the learning phase.

Learning phase – omnibus analysis

	**Likelihood of choosing the unfamiliar object**		
Predictors	*Log-Odds*	*CI*	*p*
Intercept	3.56	2.59 – 4.53	**<0.001**
Distractor compatibility - compatible vs. incompatible (ref)	-2.76	-3.92 – -1.59	**<0.001**
Age Contrast 1 - 7 vs. 5-6 year olds (ref)	0.06	-1.11 – 1.23	0.919
Age Contrast 2 - 8-10 vs. 7 year olds (ref)	-0.06	-1.10 – 0.99	0.912
Age Contrast 3 - Adults vs. 8-10 year olds (ref)	0.47	-0.28 – 1.22	0.221
Distractor compatibility x Age Contrast 1	1.38	-0.71 – 3.47	0.195
Distractor compatibility x Age Contrast 2	-1.22	-3.10 – 0.66	0.202

Distractor compatibility x Age Contrast 3	1.22	-0.13 – 2.56	0.076
**Random Effects**			
σ^2	3.29		
τ_{00} subject_id	1.97		
τ_{00} trial_id	1.32		
τ_{11} subject_id.Distractor	3.47		
τ_{11} trial_id.Distractor	1.46		
ρ_{01}	Fixed at zero		
ρ_{01}	Fixed at zero		
N subject_id	233		
N trial_id	8		
Observations	1864		

Section 2. Retention Phase – Omnibus analysis – Model Summary

Table S2. Retention phase: Model output for the logistic linear mixed-effects model predicting the likelihood of choosing the target unfamiliar object during the retention phase.

Retention phase – omnibus analysis

	Likelihood of choosing the correct unfamiliar object		
Predictors	*Log-Odds*	*CI*	*p*

Intercept	0.22	-1.27 – 1.71	0.773
Distractor compatibility - compatible vs. incompatible (ref)	0.64	-2.31 – 3.60	0.669
Choice at learning - unfamiliar vs. familiar (ref)	2.79	-9.43 – 15.01	0.654
Age Contrast 1 - 7 vs. 5-6 year olds (ref)	1.24	-4.67 – 7.15	0.682
Age Contrast 2 - 8-10 vs. 7 year olds (ref)	-0.03	-0.47 – 0.41	0.906
Age Contrast 3 - Adults vs. 8-10 year olds (ref)	0.41	0.08 – 0.75	**0.015**
Distractor compatibility x Choice at learning	-4.34	-28.78 – 20.10	0.728
Distractor compatibility x Age Contrast 1	-2.46	-14.28 – 9.36	0.684
Distractor compatibility x Age Contrast 2	0.83	0.02 – 1.63	**0.045**
Distractor compatibility x Age Contrast 3	-0.49	-1.10 – 0.12	0.118

Choice at learning x Age Contrast 1	-7.66	-56.50 – 41.18	0.759
Choice at learning x Age Contrast 2	1.74	0.21 – 3.28	**0.026**
Choice at learning x Age Contrast 3	-1.52	-2.79 – -0.26	**0.018**
Distractor compatibility x Choice at learning x Age Contrast 1	14.50	-83.15 – 112.15	0.771
Distractor compatibility x Choice at learning x Age Contrast 2	-1.17	-4.24 – 1.91	0.458
Distractor compatibility x Choice at learning x Age Contrast 3	0.68	-1.85 – 3.20	0.599
**Random Effects**			
σ^2	3.29		
$\tau_{00\ subject\_id}$	0.25		
$\tau_{00\ trial\_id}$	0.06		
$\tau_{11\ subject\_id.Distractor}$	0.36		
ρ_{01}	Fixed at zero		

ρ_{01}	Fixed at zero
$N_{subject\_id}$	233
N_{trial_id}	8
Observations	1864

Section 3. Follow-up comparisons by age group – model summary table.

Table S3. Model outputs for the logistic linear mixed-effects model predicting the likelihood of choosing the target unfamiliar object during the retention phase, separately for each age group (*p* < .05 marked in bold; ^ indicates *p* values that are above the multiple comparison corrected threshold of .0125, whereas * indicates *p* values that are below this threshold).

Retention phase - separate analyses by age group

	5-6 yo			7 yo			8-10 yo			Adults		
Predictors	*Log-Odds*	*CI*	*p*	*Log-Odds*	*CI*	*p*	*Log-Odds*	*CI*	*p*	*Log-Odds*	*CI*	*p*
Intercept	0.07	-0.24 – 0.37	0.657	0.42	0.02 – 0.83	**0.041**^	0.39	0.07 – 0.71	**0.017**^	0.86	0.65 – 1.07	**<0.001***
Distractor compatibility - compatible vs. incompatible (ref)	0.58	-0.12 – 1.28	0.104	-0.29	-0.86 – 0.28	0.316	0.53	0.04 – 1.03	**0.034**^	0.10	-0.22 – 0.42	0.546
Choice at learning -	1.00	0.12 – 1.88	**0.027**^	0.43	-0.57 – 1.44	0.396	2.02	0.89 – 3.15	**<0.001***	0.77	0.20 – 1.33	**0.008***

unfamiliar vs. familiar (ref)												
Distractor compatibility x Choice at learning	NA	NA	NA	-0.23	-2.19 – 1.73	0.819	-1.36	-3.61 – 0.88	0.233	-0.82	-1.91 – 0.27	0.141

Random Effects

σ^2	3.29	3.29	3.29	3.29
τ_{00}	0.01 subject_id	0.04 subject_id	0.06 subject_id	0.51 subject_id
	0.04 trial_id	0.17 trial_id	0.08 trial_id	0.01 trial_id
τ_{11}	1.15 subject_id.Distractor	NA	NA	0.59 subject_id.Distractor
ρ_{01}	Fixed at zero	Fixed at zero	Fixed at zero	Fixed at zero
ρ_{01}	Fixed at zero	Fixed at zero	Fixed at zero	Fixed at zero
N	29 subject_id	30 subject_id	48 subject_id	126 subject_id
	8 trial_id	8 trial_id	8 trial_id	8 trial_id
Observations	232	240	384	1008

Section 4. Retention phase: Age as a continuous predictor.

While retention accuracy increased slightly with the age of the child (Log-odds = 0.016, p = .017, CI = [0.003,0.029]), there was only a very small overall effect of distractor compatibility (Log-odds= 0.34, p =.0401, CI =[0.02, 0.67]) and no interaction with child's age (Log odds = 0.001, p = .929, CI = [-0.025, 0.028]).

Section 5. Prediction error boost effect: Comparison between Italian and English adults.

We fit the following model to the retention data from the two Italian adult samples reported here and the two English adult samples from Gambi, Pickering, et al. (2021) that used a comparable manipulation (Experiments 4 and 5):

*likelihood of choosing the correct target object ~ 1 + Distractor compatibility * Language (ref level = English) + (1+Distractor compatibility||subject_id) + (1|trial_id).*

This model revealed a significant main effect of Distractor compatibility (Log Odds = 0.29, CI = [0.06,0.52], *p* =.012) but crucially also an interaction between Distractor compatibility and Language (Log Odds = -0.59, CI = [-1.05, -0.14], *p* = .010), indicating the effect was indeed smaller in Italian than English (see Table S4). R code and English data to replicate this analysis can be found in the OSF repository (*analyses* folder, file name: *italian_english_comp_exploratory_an_adults.Rmd*).

Table S4. Comparison of adult findings in Italian (from the 2 samples reported here) and English (Experiments 4 and 5 in Gambi, Pickering, et al., 2021).

Language	Experiment	Distractor compatibility	Unfamiliar object choice at learning (%)	Target object choice at retention (accuracy) (%)
English	4 (Gambi, Pickering, et al., 2021)	incompatible	97	71
		compatible	87	80
	5 (Gambi, Pickering, et al., 2021)	incompatible	95	64
		compatible	85	76
Italian	Lab-based (current	incompatible	95	66

study)	compatible	84	65
Online (current study)	incompatible	95	71
	compatible	87	73

Section 6. Magnitude of the distractor compatibility effect on learning choices: Comparison between Italian and English adults.

The manipulation of Distractor compatibility may have been weaker for Italian-speaking participants, failing to affect their expectations as much as it did in English. To test this possibility, we compared the size of the Distractor compatibility effect in the learning phase across the Italian and English data (see Table S1). The likelihood of choosing the unfamiliar object during learning was lower in the compatible distractor condition in all experiments, and numerically very similar across languages; accordingly, the following model:

*likelihood of choosing the unfamiliar object ~ 1 + Distractor compatibility * Language (ref level = English) + (1+Distractor compatibility||subject_id) + (1+ Distractor compatibility||trial_id)*

showed no evidence for an interaction between Distractor compatibility and Language (Log Odds = 0.28, CI = [-0.79,1.35], *p* = .607), and only a main effect of Distractor compatibility across languages (Log Odds = -1.77, CI = [-3.08, -0.46], *p* = .008). Thus, it seems very unlikely that our manipulation was less successful in changing the expectations of Italian-speaking participants' compared to English-speaking participants'.

Section 7. Were Italian adults less attentive than English adults?

An alternative possibility is that Italian-speaking adults did not pay as close attention to the task as the English-speaking participants did. (Italian participants tested in the lab completed this experiment after another task, and may have been fatigued; Italian participants tested online were recruited through Prolific Academic and may have possessed a more diverse range of attentional skills compared to the undergraduate students who took part in the English experiments). Indeed, Italian-speaking participants showed numerically lower memory performance compared to English-speaking participants (see Table 7),

although this difference was not statistically reliable (Log Odds = -.26, CI = [-.55, .04], *p* = .095). Italian novel words may have been more difficult to remember than the English ones because they were longer (2 syllables instead of 1 syllable), but this did not seem to have a dramatic effect on their memory performance.

Performance on our attention checkers (four comprehension questions related to the video played during the short break between the learning and retention phases) was also very high across the two Italian adult sample (in the lab-based study, only an average of 2.57% participants responded inaccurately across the four questions; similarly, in the online study, only an average of 2.59% provided inaccurate responses).

Section 8. Correlating the size of the distractor compatibility effect and the prediction error boost effect in adults.

We computed the correlation between the size of the Choice at learning effect on retention accuracy and the size of the Distractor compatibility (i.e., prediction error boost) effect on retention accuracy across all our adult participants (from the two Italian samples reported here and Experiments 4 and 5 in Gambi, Pickering, et al., 2021). To arrive at this, we first computed the retention accuracy advantage for trials where the participant had selected the novel vs. the familiar object during the learning phase. Note that it was only possible to compute this figure for participants who chose the familiar object at least once during the learning phase: These corresponded to 58 participants across the two English studies and 62 participants across the two Italian studies. For these participants, we then also computed the difference in retention accuracy between distractor compatible and distractor incompatible trials (i.e., prediction error boost), restricted to only those trials for which they had chosen a novel object during the learning phase.

Figure S1. Scatterplot showing the relation between (x axis) the Retention advantage for words that had been explicitly associated to the novel object during the learning phase versus words that had been explicitly associated to the familiar object (more positive values indicate less attention to the wider context) and (y axis) the size of the prediction error boost effect. Separate lines represent a linear fit for Italian- and English-speaking adult participants respectively. Density plots for each group and variable are also displayed alongside the axes.

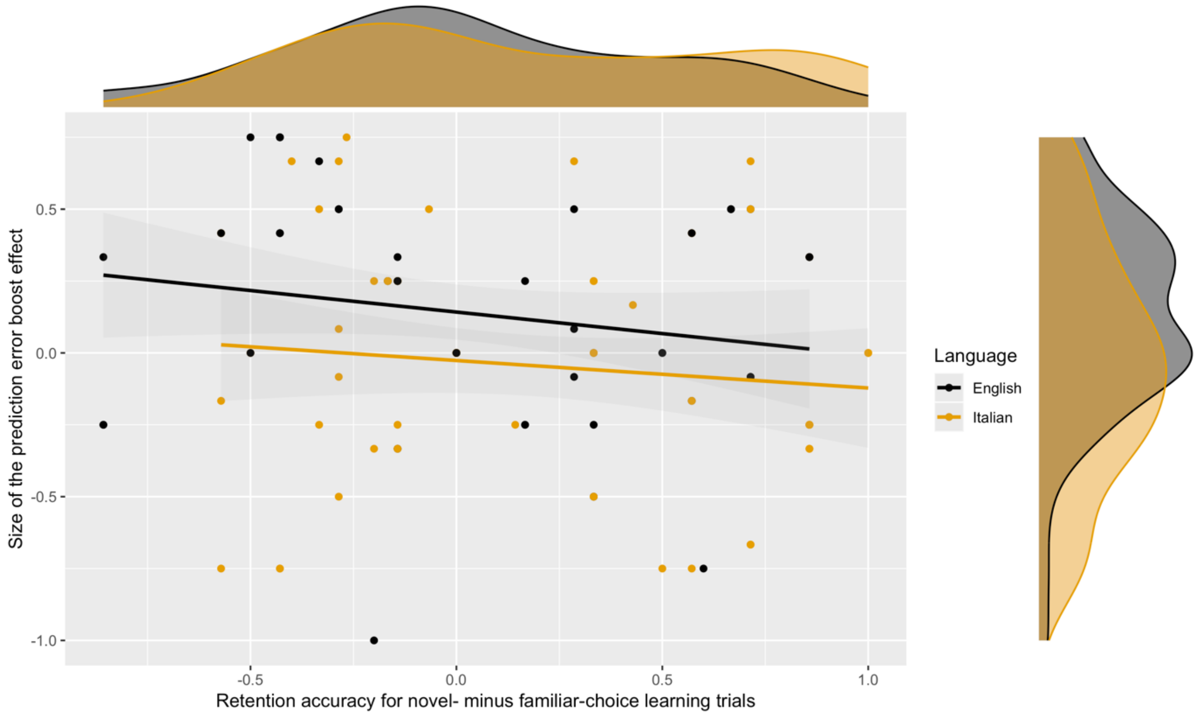

As shown in Figure S1, the data are not normally distributed, so we computed Sperman's *rho* as an estimate of the relation between the two variables. Providing some support for our hypothesis, there was a weak negative correlation, suggesting that the larger the advantage associated with choosing the unfamiliar object during learning, the smaller the prediction error boost effect (r_s = -.215, *p* = .021). Figure S1 also shows a similar trend was present in both our Italian and our English data. See the next section for the same correlational analysis on child data.

Correlating the size of the distractor compatibility effect and the prediction error boost effect in children.

Importantly, the exploratory correlational analyses reported in the previous section for adults should not be taken to suggest that sufficiently advanced context-binding abilities constitute either a necessary or a sufficient condition for the emergence of the prediction error boost during development. Indeed, 7 year olds in our study showed no memory disadvantage for trials on which they had chosen the familiar object during learning (like English adults), but they did not show a reliable prediction error boost either; conversely, 8-to-10 year olds showed both. Furthermore, there was no reliable relation between the size of the retention advantage for novel-choice learning trials and the size of the prediction

error boost effect in Italian children (see Figure S2 and OSF folder *analyses*, file name: *italian_exploratory_an_children.Rmd*), either overall or separately in each of the three age groups. We return to this point in the Discussion of the main manuscript.

Figure S2. See Figure S1 for details. Different lines represent different groups of Italian-learning children.

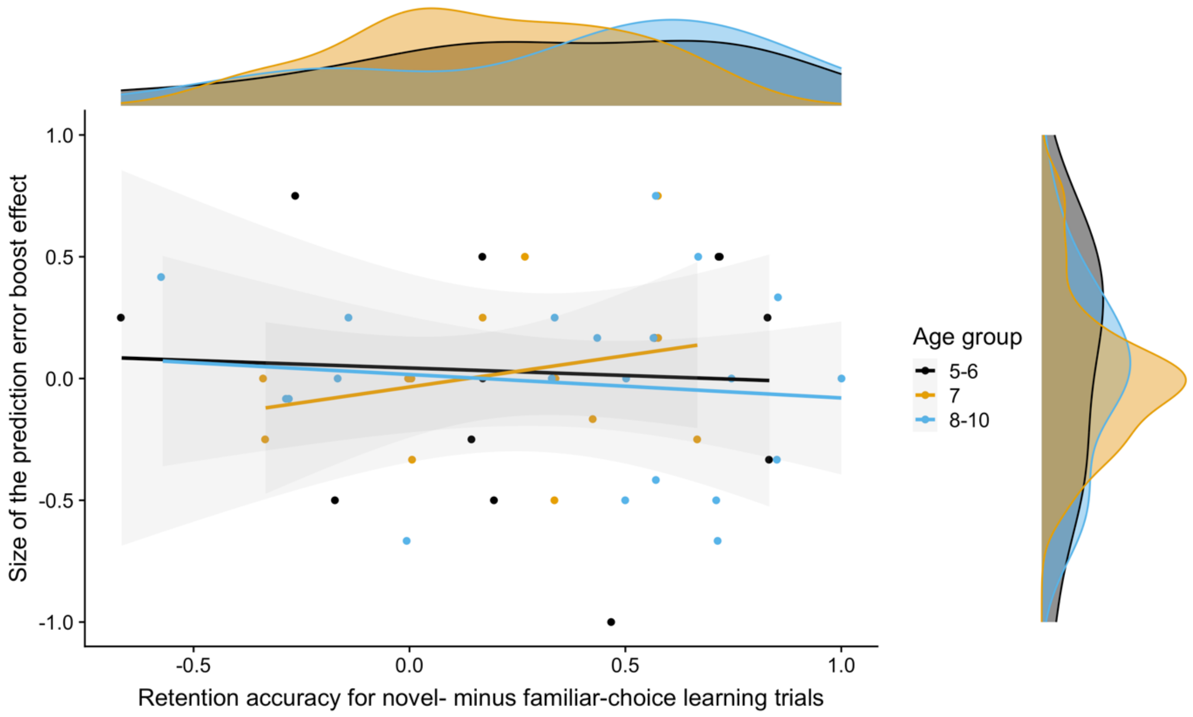

Section 9. Pooling data from English and Italian children (ages 24 to 121 months).

As mentioned in *Methods, Participants*, it is possible that our analyses for children were underpowered if the effect in children is much smaller than in adults and/or subject to greater inter-individual variability. Thus, we conducted a further exploratory analysis pooling together all English child data from Gambi, Pickering, et al., (2021) and all the Italian child data from the current study. This pooled sample covered ages from 2 to 10 years (24 to 121 months, mean age = 59 months), and the analysis accounted for the potential effects of experiment version (reference level = distractor compatibility vs. verb constraint) and

language (reference level = English vs. Italian); age was centred before being entered into the following model (see OSF folder *analyses*, file name: *it_en_child_comb_an.R*).

*Likelihood of choosing the correct target object ~ 1 + Distractor compatibility * Choice at learning * Age + Experiment + Language + (1+Distractor compatibility||subject_id) + (1 + Age||trial_id).*

There was a small (but reliable) prediction error boost effect (Log odds = 0.33, *p* = .024, CI = [0.04,0.62]), marginally qualified by an interaction with age (Log odds = 0.0106, *p* = .060, CI = [-0.0004, 0.0216]). Thus, it is possible that the effect of prediction error on word learning starts emerging towards the end of pre-school years, but it is a fragile and small effect. Interestingly, the main effect of Age was not significant (Log odds = 0.0042, p = .176, CI = [-0.0046,0.0151]), suggesting that - while children's overall memory performance in this task generally improves between the ages of 2 and 10 (see OSF folder *graphs*, file name: *plot_v3_withCI_allchildren_it_en.png*), this improvement is fairly slow and subtle.

Section 10. Bayesian analyses

Following suggestions from an anonymous reviewer, we also conducted some Bayesian analyses. These are summarised on the OSF in folder *Bayesian_analyses*, which also contains R workspaces to allow readers to reproduce our analyses as well as re-fitting our models. All Bayesian analyses were carried out in R using the package *brms* (Bürkner, 2017), version 2.20.4. First, as a sanity check, we refit the key models for the main analyses reported in manuscript using brms. The default flat priors for all fixed effect parameters were used for this purpose. The same model formula was used as for *glmer* for the analysis of learning accuracy and of retention accuracy as a function of child age in months. For the analysis of retention accuracy by age group, instead, we used a simplified model formula (not including any interactions between Distractor and Learning Accuracy) because the full model could not be estimated without warnings. As can be seen in the summary file *italian_pred_Bayesian_summary.html*, model estimates were largely consistent across frequentist and Bayesian methods and – in most cases – fixed effects that were below the .05 threshold in the frequentist analysis were associated with credible intervals that did not include 0 in the Bayesian analysis.

In a second step, we used the pooled datasets – combining data from this study with Italian participant and the English studies of Gambi, Pickering, et al. (2021) – to compute Bayes Factors (Rouder et al., 2012). For the key analysis pooling together child data from Italian and English studies, we computed the Bayes Factor for the effect of Distractor on retention accuracy. Because the *bayes_factor* function from package *brms* used bridge sampling, we followed recommendations in the literature (Rouder et al., 2012; Schad et al., 2022) and used Cauchy priors for all fixed effects. We chose uninformative priors (mean of 0 and SD of 1) for this analysis as based on previous work the evidence for an effect on children was very weak. We run this analysis 100 times to check stability, as this is a known issue with bridge sampling. While the Bayes Factor ranged from 0.22 to 10.52, the average value was 2.15 with a standard deviation of 1.62, which indicates only anecdotal evidence for an effect (Jeffreys, 1939). For the analysis pooling together adult data from Italian and English studies, we computed the Bayes Factor for the interaction between Distractor and Language on retention accuracy. Here, we repeated the analysis twice (each time with three iterations of bridge sampling), with either an uninformative Cauchy prior (mean of 0 and SD of 1) on the main effect of Distractor or a more informative Cauchy prior (mean of 0.61 and SD of 1) which assumed the effect of Distractor was equal to the one estimated from the English adult data in Experiment 4 and 5 of Gambi, Pickering, et al. (2021). In either case, the evidence in favour of the interaction was only anecdotal, though numerically the BF was slightly larger when using the more informative priors.

References

Bürkner, P. (2017). brms: An R Package for Bayesian Multilevel Models Using Stan. *Journal of Statistical Software, 80(1),* 1–28. doi:10.18637/jss.v080.i01.

Jeffreys, H. (1939). *Theory of probability.* Oxford: Clarendon Press.

Rouder, J. N., Morey, R. D., Speckman, P. L., & Province, J. M. (2012). Default Bayes factors for ANOVA designs. *Journal of Mathematical Psychology, 56(5),* 356-374.

Schad, D. J., Nicenboim, B., Bürkner, P. C., Betancourt, M., & Vasishth, S. (2022). Workflow techniques for the robust use of bayes factors. *Psychological Methods.* Advance online publication. https://doi.org/10.1037/met0000472
